# Antimicrobial activity and safety features assessment of *Weissella* spp. from environmental sources

**DOI:** 10.1002/fsn3.2885

**Published:** 2022-04-15

**Authors:** Imene Fhoula, Mohamed Boumaiza, Ghassan Tayh, Amel Rehaiem, Naouel Klibi, Imene‐Hadda Ouzari

**Affiliations:** ^1^ Laboratoire Microorganismes et Biomolécules Actives (LR03ES03) Faculté des Sciences de Tunis Université Tunis El Manar Tunis Tunisia; ^2^ Service de Microbiologie et d’Immunologie Ecole Nationale de Médecine Vétérinaire Université Manouba Sidi Thabet Tunisia; ^3^ Faculty of Medicine of Tunis Research Laboratory “Antimicrobial Resistance” LR99ES09 University of Tunis El Manar Tunis Tunisia; ^4^ Laboratory of Microbiology Charles Nicolle Hospital Tunis Tunisia

**Keywords:** antibacterial, antibiotic resistance, biogenic amines, environment, safety, *Weissella*

## Abstract

*Weissella* strains have been reported to be useful in biotechnological and probiotic determinations, and some of them are considered opportunistic pathogens. Given the widespread interest about antimicrobial susceptibilities, transmission of resistances, and virulence factors, there is little research available on such topics for *Weissella*. The aim of this study was to assess the safety aspects and antimicrobial potential of 54 *Weissella* spp. strains from different environmental sources. Antibiotic susceptibility, hemolytic activity, horizontal transfer, and antibacterial activity were studied, as well as the detection of biogenic amine BA production on decarboxylase medium and PCR was performed. All the strains were nonhemolytic and sensitive to chloramphenicol and ampicillin. Several strains were classified as resistant to fusidic acid, and very low resistance rates were detected to ciprofloxacin, tetracycline, streptomycin, lincomycin, erythromycin, and rifampicin, although all strains had intrinsic resistance to vancomycin, nalidixic acid, kanamycin, and teicoplanin. Two BA‐producing strains (*W*. *halotolerans* FAS30 and FAS29) exhibited tyrosine decarboxylase activity, and just one *W*. *confusa* FS077 produced both tyramine and histamine, and their genetic determinants were identified. Ornithine decarboxylase/*odc* gene was found in 16 of the *Weissella* strains, although 3 of them synthesize putrescine. Interestingly, eight strains with good properties displayed antibacterial activity. Conjugation frequencies of erythromycin from *Bacillus* to *Weissella* spp. varied in the average of 3 × 10^−9^ transconjugants/recipient. However, no tetracycline‐resistant transconjugant was obtained with *Enterococcus faecalis* JH2‐2 as recipient. The obtained results support the safe status of *Weissella* strains, derived from environmental sources, when used as probiotics in animal feed.

## Graphical abstract

Some *Weissella* strains presented acquired resistance, biogenic amines production, and their genetic determinants were identified. No tetracycline transconjugants were obtained with Enterococcus as recipient strain, whereas few erythromycin transconjugants were observed using Bacillus as donor. Eight strains belonging to *W*. *confusa* and *W*. *halotolerans* displayed antibacterial activity and good safety features.

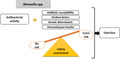

## 1 INTRODUCTION

The genus *Weissella* includes Gram‐positive heterofermentative lactic acid bacteria LAB, asporogenous short bacilli, or coccoid bacilli that can be found in pairs or short chains. Phylogenetically, bacteria within this genus belonging to the *Leuconostocaceae* family were previously grouped along with the *Leuconostoc* and *Lactobacillus* (Björkroth et al., 2009; Collins et al., 1993). Currently, 24 distinct species of *Weissella* were validated (Fusco et al., 2015; Heo et al.2019; Hyun et al., 2021; Li et al., 2020; Lin et al., 2020; Praet et al., 2015). *Weissella* spp. are broadly disturbed in a range of ecological niches where they are hypothesized to have a probiotic effect (Fusco et al., 2015), such as plants, vegetables, soil, water, and fermented foods of both plant or animal origin, as well as in feces, breast milk, animal skin and milk, and mucous membranes of humans and animals (oral, gastrointestinal tract, and vagina). Despite the fact that *Weissella* is a fairly recent genus in comparison to other LAB, it has been the subject of many studies during the past few years and has attracted the interest for use in the pharmaceutical, food, and medical sectors. It has been shown that some *Weissella* spp., specially *W*. *confusa* and *W*. *cibaria*, are able to produce exopolysaccharides EPS, mainly dextran, as natural food thickeners, and nondigestible oligosaccharides, or as prebiotics. These polymers make it of high interest for the development of applications combining EPS technological and nutritional benefits, predominantly for bakeries and the production of functional beverages (Baruah & Goyal, 2015; Juvonen et al., 2015; Korcz & Varga, 2021; Patel et al., 2012). Furthermore, the antimicrobial activity of several *Weissella* spp. has been observed against a wide range of pathogens via secondary compound production, and their potential use as probiotics has been investigated (Fhoula et al., 2018; Fusco et al., 2015; Kariyawasam et al., 2019; Trias &Bañeras, 2008; Yu et al., 2019). In relation to the health‐promoting benefits of putative probiotic *Weissella*, soe strains, primordially those belonging to *W*. *cibaria*, have been shown to have antiviral, immune‐modulating, antiobesity, anticancer, anticholesterol, and antioxidant properties (Oh & Lee, 2021; Kang et al., 2011; Park et al., 2012; Kwak et al., 2014; Fhoula et al., 2018, and Yu et al., 2019).

Despite these characteristics, the utilization of *Weissella* spp. as commercial starters or probiotics has not yet been explored. Until now, *Weissella* spp. are not generally recognized as safe (GRAS) nor as qualified presumption of safety (QPS) (Fessard & Remize, 2017). Kang et al. (2019) reported that two *W*. *cibaria* (CMU and CMS1) are commercially available as oral care probiotics in Korea, and registered as safe raw materials by the Korea Food and Drug Administration, although they have not yet been determined to be GRAS. This missing can be explained in part by the antibiotic resistance profile, biogenic amine synthesis, or infection risk (Fessard & Remize, 2017). In fact, scientists are opposed on whether or not to use *Weissella* spp., which are generally categorized as opportunistic pathogens, and occasionally linked with illnesses in people, who had weakened immune system (Fairfax et al., 2014; Fessard & Remize, 2017; Kamboj et al., 2015; Kumar et al., 2011; Teixeira, Fusieger, et al., 2021). More investigation into the safety of these strains' usage as probiotics in feed/food is required. *Weissella* spp. would have to get the safety proof to obtain GRAS accreditation through safety investigations (Fessard & Remize, 2017). Controversially, *Weissella* strains are still being used in the food and pharmaceutical industries, according to a vast number of scientific investigations (Teixeira, da Silva, et al., 2021).

This study aimed to evaluate the safety and determine the antibacterial activity of 54 *Weissella* spp. strains from distinct environmental sources in order to identify novel probiotic in foods or animal feeds. It could be used as an alternative to antibiotics, and to improve our knowledge about its safety and probiotic properties that may lead to its future use. To check in *Weissella* strains, the antibiotic resistance patterns, toxic compounds production, and any harmful genetic traits that may be transferred to other bacteria contributed to the selection of potential safety strains from novel origin.

## 2 MATERIALS AND METHODS

### 2.1 Bacterial strains and culture conditions

Fifty‐four *Weissella* strains were analyzed in this study, from different environmental origins, had previously been isolated, and molecular identified as *W*. *confusa* (*n* = 27), *W*. *halotolerans* (19), *W*. *cibaria* (03), *W*. *paramesenteroides* (03), *W*. *soli* (01) and *W*. *hellenica* (01) (Fhoula et al., 2013, 2018; Fhoula et al., 2022 (unpublished work)). *W*. *confusa* DSM‐20196 and *W*. *cibaria* DSM‐15878 were involved as type strains. Weissella strains were maintained on de Man Rogosa and Sharpe (MRS) broth (Fluka, Milan, Italy) at 30 or 37°C for 24h. The strains were then used for the safety assessment. The following were used as indicators strains, including *Escherichia coli* DH5α, *Listeria monocytogenes* L15, *Salmonella* typhimurium IPT13, *Enterococcus faecalis* ATCC 29,212, *Pseudomonas aeruginosa* ATCC 27,853, *Paenibacillus larvae*, and *Staphylococcus aureus* ATCC 6538, and were grown in brain–heart infusion (BHI) broth (Biolife) at 37°C. *Bacillus thuringiensis* subsp. *Kurstaki*, gram‐positive model plasmid, harboring conjugative pAW63 (*Erm B*), resistant to streptomycin and erythromycin (STR^r^ ERY^r^), was chosen as donor to tested *Weissella* strains, and inoculated in Elliker medium, containing 2% glucose as sugar, with corresponding antibiotics. *E*. *faecalis* JH2–2, plasmid‐free recipient, resistant to rifampicin and fusidic acid (RIF ^r^, FUS^r^, and VAN^s^), was used as a standard recipient with selected *Weissella* strains, and was grown in BHI medium containing the appropriate antibiotics for 24h at 37°C. Transconjugant (TCs) strains of *Weissella* (NAL^r^ ERY^r^) were selected on Elliker agar medium supplemented with nalidixic acid and erythromycin for 48 h at 30°C. TCs of *E. faecalis* JH2‐2(tet) and JH2‐2(van) were detected on BHI agar supplemented with fusidic acid and rifampicin, and tetracycline or vancomycin, respectively, for 48 hr at 30°C or 37°C. Antibiotics (Sigma, Aldrich) were used at the following concentrations per ml: nalidixic 15 µg, fusidic acid 25 µg, rifampicin 50 µg, erythromycin 5 µg, tetracycline 10 µg, and vancomycin 30 µg.

### 2.2 Antibiotic susceptibility testing and MIC Determination

Susceptibility to 13 antibiotics was performed by disk diffusion method as recommended by the standard criteria Clinical and Laboratory Standards Institute (CLSI, 2018) and Comite de l’Antibiogramme de la Societe Francaise de Microbiologie (CA‐SFM, 2013). The analysis was carried out on modified Muller Hinton MH agar (with 2% glucose and 0.4% yeast extract) plates to assure accurate growth of all the *Weissella* strains. The used antibiotic discs (Bio‐Rad Laboratories, Hercules, CA, USA) were ampicillin (10 µg), chloramphenicol (30 µg), rifampicin (5 μg), erythromycin (15 μg), lincomycin (2 μg), tetracycline (30 μg), ciprofloxacin (5 µg), fusidic acid (10 µg), vancomycin (30μg), nalidixic acid (30 µg), kanamycin (30 µg), teicoplanin (30 µg), and streptomycin (10 µg). High‐level of aminoglycoside resistance was detected using high‐charge disk of streptomycin (300 μg). A 0.5 McFarland turbidity standard inoculum from overnight strains was inoculated onto the surface of modified MH agar plates. After incubation at 37°C for 24 h, the inhibition zone size was interpreted as sensitive (S), intermediate (I), and resistant (R) to the antimicrobial agent.

Minimum inhibitory concentration MIC for tetracycline, chloramphenicol, and erythromycin (range: 0.5–512 μg/ml) was determined for *Weissella* strains exhibiting intermediate sensitivity or resistance to the three antibiotics described above by broth microdilution method according to the International Standard ISO10932/IDF 223 (ISO, 2010), using modified MHB. The microplates with different antimicrobial agents were added with bacterial inoculum to a turbidity of 0.5 McFarland scale, and then incubated anaerobically at 37°C for 24 h. The experiment was performed twice, each in triplicate. For *Weissella,* there are currently no EFSA’s cut‐off values. MICs were compared to the epidemiological cut‐off (ECOFF) values adopted for *Leuconostoc* spp. from the European Food Safety Authority EFSA (EFSA Panel on Additives and Products or Substances used in Animal Feed (FEEDAP) et al., 2018), and for *Weissella* spp. by Suhonen (2019), to distinguish between susceptible and resistant strains. All MIC testing was performed in duplicates. *E*. *faecalis* ATCC29212 was used as quality control for antibiotic susceptibility testing by disc diffusion and MIC. All antimicrobial agent powders were obtained from Sigma‐Aldrich.

### 2.3 Hemolytic activity

Hemolytic activity was determined by streaking bacterial cultures on Columbia agar plates supplemented with 5% of horse blood (bioMérieux) and then hemolysis zones around the colonies were checked (Maragkoudakis et al., 2006). All experiment was performed in three replicates.

### 2.4 Antibacterial activity against pathogen and food‐borne bacteria

The antibacterial activity was determined using the agar well‐diffusion method described by Tagg and McGiven (1971). Seven indicator strains were used to assess the growth inhibition activity of *Weissella* strains, involving *S*. *aureus* ATCC 6538, *L*. *monocytogenes* L15, *Pa. larvae*, *P*. *aeruginosa* ATCC 27,853, *E*. *faecalis* ATCC 29,212*, S*. typhimurium IPT,13, and *E*. *coli* DH5α. The cell‐free supernatants (CFS) derived from broth cultures (48h) of all *Weissella* strains were tested. To remove the effects of organic acid and hydrogen peroxide, the supernatants were treated with catalase (300 IU/ml, 37°C, 1 h, Sigma Aldrich) and neutralized with 1 M NaOH. These catalase‐treated cell‐free neutralized supernatants were then examined for antimicrobial activities, including as those due to bacteriocins. All indicator strains were grown in BHI broth at 37°C. Trypticase soy agar plates were overlaid with 5 ml of soft agar (0.75%) containing 50 μl of freshly grown culture. The wells were made in agar and filled with 100 μl of CSF of tested strain. After incubation at 37°C for 18 h, the diameter of the inhibition zones was measured. All antibacterial tests were performed in triplicate.

### 2.5 Detection of potential biogenic amine producer

The amino acid decarboxylase activity of *Weissella* strains was assessed in the decarboxylase agar medium, as described by Bover‐Cid and Holzapfel (1999), containing 1% of the appropriate precursor amino acids l‐tyrosine, l‐histidine, and l‐ornithine hydrochloride (Sigma). The tested strains were spotted on the decarboxylase agar medium and incubated anaerobically at 37°C for 72 h. The presence of a purple color in the medium around the colonies indicated a positive reaction; however, a yellow color indicated a negative reaction.

### 2.6 DNA extraction

Genomic DNA extraction of *Weissella* strains was performed enzymatically using a kit InstaGene^TM^ Matrix (BioRad) according to the manufacturer's instructions.

### 2.7 PCR detection of decarboxylase‐related genes

PCR reactions were performed to detect the occurrence of tyrosine (*tdc*), histidine (*hdc*), and ornithine (*odc*) decarboxylase genes with the respective primers Tdc1/Tdc2, JV16HC/JV17HC, and ODC1F/ODC2R and conditions as previously described (Costantini et al., 2006; Fernández et al., 2004; Le Jeune et al., 1995). Reaction mixture (25 µl) contained 200 ng of bacterial DNA, 0.2 mM of each dNTP, 1 µM of each primer, 1.5 mM of MgCl_2_, 1× Buffer, and 1 U *Taq* DNA polymerase (Fermentas). *W*. *confusa* DSM*‐*20196 was used as a positive control for *odc* gene, and *En*. *faecium* MMRA (Rehaiem et al., 2012) for the *hdc* and *tdc* genes. PCR products were resolved in 2% (w/v) agarose gel and visualized under UV transillumination.

### 2.8 Detection of antibiotic resistance genes

The strains displaying acquired antibiotic resistance(s) to erythromycin, tetracycline, and chloramphenicol were inspected by PCR for resistance genes [*erm*(B)], [*cat*], and [*tet*(M), *tet*(O), *tet*(L), *tet*(S), and *tet*(k)], respectively (Aarestrup et al., 2000; Sutcliffe et al., 1996). PCR amplification was performed as previously described (Rizzotti et al., 2005, 2009).

### 2.9 Transfer of antibiotic resistance

The transferability of erythromycin resistance of the *B*. *thuringiensis* sv *kurstaki* strain (Sm^R^ Ery^r^), potential donor, was evaluated using three recipient strains (*W*. *halotolerans* V10, *W*. *paramesenteroides* FS45, and *W*. *confusa* FS53) that are sensitive to erythromycin but resistant to nalidixic acid. To assess the transferability of tetracycline resistance of the two *W*. *confusa* (FS44 and FS63) obtained from olive rhizosphere soil, *E. faecalis* JH2‐2 (FUS^r^, RIF^r^, and TET^s^), free from plasmids, was chosen as the recipient strain. The filter mating procedure was used to investigate antibiotic resistance transfer, as reported by Gevers et al. (2003). Briefly, donor and recipient cell cultures (V/V of 1 ml), at exponential growth, were mixed and deposited onto a sterile nitrocellulose membrane filter (0.45 µm pore size, Milli‐pore, USA), and the filter was incubated on nonselective medium agar based on the ideal growth conditions of the recipient strain. The bacteria were rinsed off the filters and suitable dilutions were seeded onto donor‐, recipient‐, and TC‐selective agar plates. Three replicates of all matings were conducted.

## 3 RESULTS

### 3.1 Antimicrobial‐resistant profiles and genetic determinants

Tables 1 and 2 summarize the prevalence and antibiotic resistance phenotypes perceived among tested *Weissella* strains based on the disk diffusion method. We recorded a high prevalence of resistance to fusidic acid in 48.1% of *Weissella* strains while a low resistance rate was observed to ciprofloxacin 14.8%, tetracycline 11.1%, streptomycin (high‐level resistance) 7.4%, lincomycin 7.4%, and rifampicin 7.4%. All the tested strains were susceptible to ampicillin, chloramphenicol, and erythromycin, while resistant to vancomycin, teicoplanin, nalidixic acid, kanamycin, and streptomycin (low‐level resistance). Intermediate resistance to rifampicin was seen in seven strains (13%), for lincomycin and erythromycin in three strains (5.6%), and for chloramphenicol in two strains (3.7%). *W*. *confusa* LV30 and LV20 (from plants) and FS44 (from rhizosphere soil) were the only strains that displayed intermediate resistance to erythromycin; among them the last two strains cited showed moderate resistance to chloramphenicol. *W*. *soli* F96, *W*. *halotolerans* (FAS27 and FS058), and *W*. *confusa* (FS044, FS063, and LV20) demonstrated resistance to tetracycline. None of the 54 *Weissella* spp. strains showed hemolytic activity.

**TABLE 1:** Prevalence of antibiotic resistance in *Weissella* isolates from environmental sources using disk diffusion assay

Antibiotics used		No. of resistance	No. of intermediate	No. of sensitive
Teicoplanin	30 μg	54 (100%) I.R	‐	–
Kanamycin	30 μg	54 (100%) I.R	‐	–
Nalidixic acid	30 μg	54 (100%) I.R	–	–
Vancomycin	30 μg	52 (98%) I.R	–	–
Streptomycin	10 μg	46 (85.2%) I.R	–	8 (14.8%)
Fusidic acid	10 μg	26 (48.1%)	–	28 (51.8%)
Ciprofloxacin	5 μg	8 (14.8%)	–	46 (85.2%)
Tetracyclin	30 μg	6 (11.1%)	–	48 (88.9%)
Streptomycin	300 μg	4 (7.4%)	–	50 (92.6%)
Lincomycin	2 μg	4 (7.4%)	3 (5.6%)	47 (87%)
Erythromycin	15 μg	–	3 (5.6%)	51 (94.4%)
Ampicillin	10 μg	–	–	54(100%)
Rifampicin	5 μg	4 (7.4%)	7(13%)	43(79.6%)
Chloramphenicol	30 μg	–	2(3.7%)	52 (98%)

Note

The numbers in parentheses represent the proportion of resistant strains among the tested strains.

Abbreviations: I.R, intrinsic resistance. No, number.

**TABLE 2:** Preliminary safety evaluation of *Weissella* spp. strains

Species	Strains^a^	Origin	Antibiotic resistance phenotype^b^	Virulence factors		Hemolytic activity
				Phenotype^c^	Genotype	
*W*. *confusa*	FS066, FS004	Rhizospheric soil	CIP, FUS, STR	Hdc^−^, Tdc^−^, Odc^−^	‐	–
	FS052		CIP, FUS, LIN, STR	Hdc^−^, Tdc^−^, Odc^+^	*odc^+^*	–
	FS053		CIP, STR, LIN^f^, RIF^f^	Hdc^−^, Tdc^−^, Odc^+^	*odc^+^*	–
	FS063		CIP, FUS, STR, TET, RIF^f^	Hdc^−^, Tdc^−^, Odc^−^	–	–
	FS076		FUS, STR	Hdc^−^, Tdc^−^, Odc^−^	–	–
	FS077		FUS, STR	Hdc^+^, Tdc^+^,Odc ^−^	*hdc* ^+^, *tdc^+^*, *odc* ^+^	–
	FS036, FS061		FUS, STR	Hdc^−^, Tdc^−^, Odc ^−^	*odc^+^*	–
	FS054		FUS, STR	Hdc^−^, Tdc^−^, Odc^−^	–	–
	FS044		STR, TET, ERY^f^_,_ CHL^f^	Hdc^−^, Tdc^−^, Odc^−^	–	–
	LV30	Desert plants	CIP, FUS, STR_,_ RIF, TET, ERY^f^	Hdc^−^, Tdc^−^, Odc^−^	–	–
	LV9		FUS, STR	Hdc^−^, Tdc^−^, Odc^−^	–	–
	LV2			Hdc^−^, Tdc^−^, Odc^−^	–	–
	LV11		STR^d^	Hdc^−^, Tdc^−^, Odc ^−^	*odc^+^*	–
	LV29			Hdc^−^, Tdc^−^, Odc ^−^	*odc^+^*	–
	LV20		STR^d^, TET, ERY^f^_,_ CHL^f^	Hdc^−^, Tdc^−^, Odc^−^	–	–
	LV28		STR, RIF	Hdc^−^, Tdc^−^, Odc^−^	–	–
	LV31		CIP, STR, RIF^f^	Hdc^−^, Tdc^−^, Odc^−^	–	–
	LF42	Camel feces	FUS, STR	Hdc^−^, Tdc^−^, Odc^−^	–	–
	LF77		FUS, LIN, STR	Hdc^−^, Tdc^−^, Odc^−^	–	–
	LF7		STR, RIF^f^	Hdc^−^, Tdc^−^, Odc^−^	–	–
	LF9		STR^d^, RIF	Hdc^−^, Tdc^−^, Odc ^−^	*odc^+^*	–
	LF80		STR, RIF^f^	Hdc^−^, Tdc^−^, Odc^−^	–	–
	FJ2	Ants' gut	FUS, STR	Hdc^−^, Tdc^−^, Odc^+^	*odc^+^*	–
	FAS5		FUS, STR	Hdc^−^, Tdc^−^, Odc^−^	–	–
	FAS23^e^		STR, RIF^f^	Hdc^−^, Tdc^−^, Odc^−^	–	–
*W*. *halotolerans*	FAS17	Ants' gut	LIN, CIP, FUS, STR	Hdc^−^, Tdc^−^, Odc^−^	–	–
	FAS42;FAS16; FAS18		FUS, STR	Hdc^−^, Tdc^−^, Odc^−^	–	–
	FAS3		FUS, STR, LIN^f^	Hdc ^−^, Tdc^−^, Odc ^−^	*hdc^+^*, *odc^+^*	–
	FAS22		FUS, STR	Hdc ^−^, Tdc^−^, Odc^−^	*hdc^+^*	–
	FAS28		FUS, STR,	Hdc^−^, Tdc^−^, Odc ^−^	*odc^+^*	–
	FAS31		STR	Hdc^−^, Tdc^−^, Odc^−^	–	–
	FAS65		FUS, STR	Hdc^−^, Tdc^−^, Odc^−^	–	–
	FAS15; FAS24		STR	Hdc^−^, Tdc^−^, Odc ^−^	*odc^+^*	–
	FAS29		STR, LIN	Hdc^−^, Tdc^+^, Odc ^−^	*tdc^+^*, *odc* ^+^	–
	FAS30			Hdc^−^, Tdc^+^, Odc^−^	*tdc^+^*	–
	FAS27		STR, TET, RIF^f^	Hdc^−^, Tdc^−^, Odc ^−^	*odc^+^*	–
	V5; LV10	Desert plants	STR	Hdc^−^, Tdc^−^, Odc^−^	–	–
	LV27			Hdc^−^, Tdc^−^, Odc^−^	–	–
	LF99	Camel feces	FUS, STR	Hdc^−^, Tdc^−^, Odc^−^	–	–
	FS058	Rhizospheric soil	STR^d^	Hdc^−^, Tdc^−^, Odc^−^	–	–
*W*. *paramesenteroides*	FS045^e^		STR, RIF	Hdc^−^, Tdc^−^, Odc^−^	–	–
	FS060			Hdc^−^, Tdc^−^, Odc ^−^	*odc^+^*	–
	FS064		FUS, STR	Hdc^−^, Tdc^−^, Odc^−^	–	–
*W*. *cibaria*	FAS7	Ants' gut	FUS	Hdc^−^, Tdc^−^, Odc^−^	–	–
	LF81	Camel feces		Hdc^−^, Tdc^−^, Odc ^−^	*odc^+^*	–
	LF67			Hdc^−^, Tdc^−^, Odc^−^	–	–
*W*. *hellenica*	LF4			Hdc^−^, Tdc^−^, Odc^−^	–	–
*W*. *soli*	LF96		STR, TET, LIN^f^	Hdc^−^, Tdc^−^, Odc ^−^	*odc^+^*	–
*W*. *confusa*	DSM−20196	Sugar cane	STR, RIF	Hdc^−^, Tdc^−^, Odc ^−^	*odc^+^*	–
*W*. *cibaria*	DSM−15878	Chili bo	RIF^f^, TET^f^, ERY^f^	Hdc^−^, Tdc^+^, Odc^−^	*tdc^+^*	–

^a^ All the tested *Weissella* strains are resistant to vancomycin (VAN), nalidixic acid (NAL), kanamycin (KAN), and teicoplanin (TEC).

^b^ Abbreviation of antibiotics: TET, tetracycline; LIN, lincomycin; CHL, chloramphenicol; RIF, rifampicin; CIP, ciprofloxacin; ERY, erythromycin; FUS, fusidic acid.

^c^ Hdc, Tdc, and Odc refer to histidine, tyrosine, and ornithine decarboxylase activity, respectively.

^d^ High level of resistance to streptomycin (300 µg).

^e^ Vancomycin sensitive.

^f^ Intermediate resistance.

MIC ranges of three selected antibiotics were performed for six strains resistant or intermediate resistance to tetracycline, erythromycin, and chloramphenicol. The results obtained are presented in Table 3. The breakpoint values were not described for *Weissella*, heterofermentative *Leuconostoc*‐like LAB (Collins et al., 1993); hence, it was categorized as resistant to a specific antibiotic if the MIC value (mg/L) was higher than the breakpoints given for *Leuconostoc* by EFSA (EFSA Panel on Additives and Products or Substances used in Animal Feed (FEEDAP) et al., 2018), according to the cut‐off levels proposed by Jeong and Lee (2015) and Suhonen (2019) for *Weissella*. To elucidate the mechanism responsible for the resistance phenotypes perceived, genes encoding those phenotypes were checked by PCR as described above (Table 2).

**TABLE 3:** Minimum inhibitory concentration (MIC) distributions of tetracycline, erythromycin, and chloramphenicol for selected *Weissella* strains from different origins, determined by the broth microdilution method

Tested strain		Source of isolation	Susceptibility to the following antibiotic MIC (mg/L)				Resistance gene(s)
			VAN	TET	ERY	CHL	
ECOFF (µg/ml) *Leuconostoc* ^a^			IR	8	1	4	
*Weissella* ^b^			IR	8	4	12/16	
*W*. *confusa*	FS44	Rhizospheric soil	*n*.r.	256	16	32	*tet*(K), *tet*(S)
	FS63		*n*.r.	>256			*tet*(M)
	V20	Desert plants	*n*.r.	>256	16	32	*tet*(K)
	V30		*n*.r.	32	16		*tet*(M), *tet*(O)
*W*. *halotolerans*	FAS27	Ants' gut	*n*.r.	256			*tet*(K), *tet*(S)
*W*. *soli*	F96	Camel feces	*n*.r.	256			*tet*(K)

TET = tetracycline; ERY = erythromycin; CHL = Chloramphenicol.

^a^ For *Weissella* strains, epidemiological cut‐off (ECOFF) values were not described, the breakpoint values suggested by EFSA (EFSA Panel on Additives and Products or Substances used in Animal Feed (FEEDAP) et al., 2018) for the genus *Leuconostoc* were considered.

^b^ The breakpoint values according to Suhonen (2019) are given. LAB with a MIC value higher than the ECOFF or breakpoints of diverse recommendations reported are considered resistant strains; IR, intrinsically resistant; *n*.r., not required.

The chloramphenicol MIC values (32 mg/L) obtained for the two *W*. *confusa* (FS44 and V20) were higher than the recommended breakpoint value (4–12/16 mg/L). No *cat* gene encoding chloramphenicol acetyltransferase has been detected in these strains (Table 3). Erythromycin‐resistant *W*. *confusa* (FS44, V20, and V30), isolated from environment or rhizosphere soil and desert plants, displayed MIC value of 16 mg/L; however, no resistance determining gene (*erm*B) was detected (Table 3). All the six strains were found to be resistant to tetracycline with MICs≥256 mg/L, except *W*. *confusa* V30 (32 mg/L). In this study, we similarly verified the presence of tetracycline resistance genes *tet*(K), *tet*(L), *tet*(M), and *tet*(S). Resistance genes were detected in 100% of tetracycline‐resistant strains. It was associated with the presence of the resistance genes *tet*(K), *tet*(S), *tet*(M), and *tet*(O) (Table 3). Combinations of tetracycline resistance genes were not common, with two tetracycline‐resistant strains carrying more than one resistance gene. Four tetracycline‐resistant strains harbored the tetracycline efflux gene *tet*(K), among them *W*. *confusa* FS63 and *W*. *halotolerans* FAS27 stains contained also the *tet*(S) gene encoding a ribosomal protection protein. Besides, other genes encoding ribosomal protection proteins [*tet*(M) and *tet*(O)] were detected in *W*. *confusa* FS44 and *W.confusa* V30.

### 3.2 Biogenic amine production of *Weissella*

The presence of BA‐producing *Weissella* was qualitatively investigated by assessing color variations in the decarboxylase medium. Histidine decarboxylase, ornithine decarboxylase, and tyrosine decarboxylase were screened. Decarboxylase‐positive bacteria produce alkaline amines, inducing pH change rounding colonies (Bover‐Cid & Holzapfel, 1999). Table 2 showed the phenotypic expression of tyramine, histamine, and putrescine via tyrosine, histidine, and ornithine decarboxylase activities, respectively, by six *Weissella* strains. We detected putrescine production via odc pathway in three *W*. *confusa* (FS52 and FS53) from rhizosphere soil, and (FJ2) from ant's gut. Tyramine was generated by three strains (5.6%) and histamine production by one strain (1.9%). Two BA‐producing *W*. *halotolerans* strains (FAS30 and FAS29) exhibited tyrosine decarboxylase activity. However, one *W*. *confusa* (FS77) produced two types of BA, tyramine and histamine.

### 3.3 Detection of genes encoding histidine, tyrosine, and ornithine decarboxylases

To examine the presence of genes *hdc*, *tydc*, and *odc* in the 54 *Weissella* strains, which could reveal or not the BA production ability in the decarboxylase medium, we performed PCR amplification investigation. The results showed that decarboxylase‐related gene *odc* was determined in 16 strains (29.6%), of which only three strains expressed phenotypically putrescine production. On the other hand, BA gene *tydc* was proved in three initially tyramine‐producing strains *W*. *halotolerans* (FAS30 and FAS29) and *W*. *confusa* FS77. Besides, BA gene *hdc* was determined only in histamine‐producing *W*. *confusa* FS77.

### 3.4 Antagonistic activity

Antibacterial screening of 54 *Weissella* strains was characterized by high, medium, and no significant inhibition activity against the indicator pathogens, including *Pa. larvae, L. monocytogenes, E. faecalis*, *S*. *aureus, E. coli*, *S*. *typhimurium,* and *P. aeruginosa* (Table 4). The sensitivity of the indicator strains was assessed based on the diameter (mm) of growth inhibition zones. The majority of the tested strains were able to inhibit the growth of Gram‐positive and ‐negative indicator strains (*p* < .05), with the greatest zones of inhibition for *P*. *aeruginosa* (36 ± 1.8 mm) followed by *S. aureus* (34 ± 1.6 mm), *E*. *coli* (26 ± 0.9 mm), *E. faecalis* (26 ± 1 mm), *Pa. larvae* and *S*. *typhimurium* (24 mm), and *L*. *monocytogenes* (20 ± 1.6 mm).

**TABLE 4:** Antibacterial activity of *Weissella* spp. strains

*Weissella* spp.	Strains	*Paenibacillus larvae*	*Listeria monocytogenes* LM15	*Staphylococcus aureus* ATCC 6538	*Enterococcus faecalis* ATCC 29,212	*Pseudomonas aeruginosa* ATCC 27,853	*Escherichia coli* DH5α	*Salmonella typhimurium* IPT13
*W*. *confusa*	FS066	+	++	+	+	+	+	+
	FS004	++	−	−	−	+++	+++	+++
	FS052	+	++	+++	++	+++	++	+++
	FS053	+++	−	−	−	+	++	+
	FS063	−	+	−	−	+++	+	+++
	FS076	++	+	−	+	+++	++	+++
	FS077	++	−	++	++	++	++	++
	FS036	++	+	+++	−	+++	−	+++
	FS054	++	+	+++	−	+++	+++	+++
	FS061	−	+	−	−	+++	+	+++
	FS044	++	++	++	−	++	+++	−
	LV30	+	+	−	+	+	+	+
	LV9	−	−	++	−	−	+++	−
	LV2	+++	−	++	+	+	+	+
	LV5	+	+	+++	++	−	++	++
	LV11	−	+	++	++	−	++	−
	LV20	+++	−	+	+	++	+	++
	LV27	+	++	+	++	++	++	++
	LV29	++	+	+	++	+	++	++
	LV28	++	+++	++	++	++	+++	++
	LV31	−	+	+++	−	++	+++	++
	LF42	+	−	++	+++	−	−	−
	LF77	+++	++	+++	−	+++	+++	++
	LF7	−	−	−	+	−	−	−
	LF9	−	++	++	+	−	−	−
	LF80	−	−	−	+	++	−	++
*W*. *confusa*	FJ2	−	−	−	−	−	−	+++
	FAS5	−	−	−	+++	−	−	++
	FAS23	−	+	−	+	−	−	++
*W*. *halotolerans*	FAS17	+++	+++	++	++	++	+++	+
	FAS42	+	−	−	−	+	−	−
	FAS3	−	−	−	−	−	−	−
	FAS16	++	−	−	−	+	−	−
	FAS18	++	+	++	−	++	+++	++
	FAS22	−	−	−	−	++	−	−
	FAS28	++	−	+++	++	+	++	+
	FAS30	−	−	−	++	+	+	−
	FAS65	−	−	+	−	−	−	−
	FAS15	−	−	−	−	−	−	−
	FAS24	−	−	−	−	++	−	−
	FAS29	++	−	++	++	+	−	−
	FAS31	+++	++	+++	++++	++++	+	+++
	FAS27	−	−	++	++	+++	+++	+++
	LV10	+	+++	+++	+++	++	++	+
	LF99	−	+	+	+	+++	+++	+++
	FS058	+	−	−	+++	−	−	−
*W*. *paramesenteroides*	FS045	++	+	+	+	+	−	+
	FS060	+	−	−	++	−	−	−
	FS064	++	++	+	++	+	++	+
*W*. *cibaria*	FAS7	−	−	−	+	−	++	++
	LF81	−	−	−	++	−	−	−
	LF67	−	−	++	+++	−	−	−
*W*. *hellenica*	LF4	++	−	+	++	−	−	−
*W*. *soli*	LF96	++	−	−	−	−	−	−

Note

(*d*) Inhibition zone diameter: (+), weak (*8 ≤ d <13*); (++), medium (*13 ≤ d <18*); (+++), and high (*18 ≤ d*) antibacterial activity.

The results showed that 28 strains (51.9%) have significant inhibition against one or more pathogens, with high inhibitory activity. The highest inhibitory effect of Weissellas was observed against each of the three gram‐negative pathogens (around 20% and 22%), after that in the case of *S. aureus* (17%), followed by *Pa*. *larvae* (11%), *E. faecalis* (9%), and *L. monocytogenes* (6%). This activity was recorded for the species of *W*. *confusa*, then in *W*. *halotolerans*. *W*. *confusa* (FS66, FS52, F77, and V28) and *W*. *halotolerans* (FAS17, FAS31, and V10) demonstrated inhibitory activity against all the tested pathogens. *W*. *confusa* FS076 had top growth inhibitory activity against *P. aeruginosa*. *W*. *halotolerans* FAS27 had the highest inhibitory activity against *S*. *aureus*, *S*. Typhimurium, and *E. coli*; *W*. *cibaria* F67 showed also strong inhibitory activity toward *E*. *faecalis*. *W*. *confusa* F77 had strong activity against *Pa. larvae* with ZI 24 ± 0.6 mm. Anti‐*Listeria* activity was shown to be really high for *W*. *halotolerans* (FAS17 and V10) and *W*. *confusa* V28. The very anti‐*Staphylococcus* activity was recorded for *W*. *confusa* (FS52, FS36, FS54, V5, V31, and F77) and *W*. *halotolerans* (FAS28, FAS31, and V10).

### 3.5 Transferability of antibiotic resistance genes ARGs

The ability of donors to transmit antibiotic resistance to the recipients was tested by filter mating approach. As shown in Table 5, no colonies of presumptive transconjugants (RIF^r^ and TET^r^) were observed after mating of the two tetracycline‐resistant *W*. *confusa* (FS44 and FS63) donor strains with *Enterococcus* JH2‐2 as a recipient on the selective agar plates. However, *Bacillus thuringiensis* could transfer their conjugal plasmid *p*AW63 (erythromycin resistance) to three tested *Weissella* spp. as recipients, at frequencies around 3 × 10^−9^ transconjugants per recipient (Table 5).

**TABLE 5:** Frequency of pAW63 (erythromycin resistance) and tetracycline transfer from donors *Bacillus thuringiensis* and two *Weissella confusa* strains, respectively, to the corresponding recipients (CFU/ml)

Donors		Recipients	Transfer frequency (no. of transconjugants/recipient)^a^
*B. thuringiensis* subsp. *kurstaki*	*W*. *paramesenteroides* FS45		1.8 × 10^−9^
	*W*. *halotolerans* V10		7 × 10^−9^
	*W*. *confusa* FS53		1.2 × 10^−10^
*W*. *confusa* FS44	*En. faecalis* JH2−2		–
*W*. *confusa* FS63	*En. faecalis* JH2−2		–

^a^ Transfer frequency is expressed as the number of transconjugants/number of recipient cells; results represent the mean of three experiments.

## 4 DISCUSSION

Until today, the investigation of antibacterial resistance profiles of the genus *Weissella* is limited to few published reports (Akpınar Kankaya & Tuncer, 2020; Ayeni et al., 2011; Fhoula et al., 2018; Jeong & Lee, 2015; Lee et al., 2012; Muñoz‐Atienza et al., 2013; Patel et al., 2014). In this context, antimicrobial susceptibility testing can be used as required selection criterion for probiotic cultures as well as a useful guide for precise antibiotic therapy. Our study highlights for the first time the antibiotic resistance for *W*. *halotolerans, W*. *paramesenteroides*, *W*. *soli,* and *W*. *hellenica* in addition to *W*. *confusa* and *W*. *cibaria*. All of the resistance that has been found is strain dependent. Similarly, to the findings of Jeong and Lee (2015), *Weissella* strains have been shown to be susceptible to ampicillin (inhibitor of cell wall synthesis) and chloramphenicol (inhibitor of proteins synthesis), but not to erythromycin or tetracycline.

As a result, we deduced resistance to nalidixic acid, kanamycin, streptomycin, and teicoplanin, as well as vancomycin, which can be attributed to intrinsic resistance in the *Weissella* genus. Nevertheless, susceptibility seems to be species and strain dependent. According to Abriouel et al. (2015), resistance to high concentrations of vancomycin appears to be a widespread trait in the genera *Weissella* and *Leuconostoc*. Weissellas are recognized as intrinsically resistant to antibiotics inhibiting cell wall biosynthesis (glycopeptides) like vancomycin, comparable to other *Lactobacillus*, *Pediococcus*, and *Leuconostoc* species (Akpınar Kankaya & Tuncer, 2020; Ammor et al., 2007; Danielsen & Wind, 2003; Gueimonde et al., 2013; Muñoz‐Atienza et al., 2013). Therefore, this resistance cannot be associated with acquired resistance genes (Abriouel et al., 2015). On the other hand, resistance to teicoplanin, one of the glycopeptides, was also revealed in the genome of some Weissellas due to the presence of the *vanZ* resistance gene as it is with *W*. *confusa* LBAE C39‐2, *W*. *cibaria* KACC 11,862, and *W*. *paramesenteroides* ATCC 33,313 (Abriouel et al., 2015). However, in our study, this resistance was detected in all the tested *Weissella* strains for six different species (*W*. *confusa*, *W*. *cibaria*, *W*. *paramesenteroides*, *W*. *halotolerans*, *W*. *soli,* and *W*. *hellenica)*. Additionally, kanamycin resistance in *Weissella* strains was observed, correlating with prior findings in food‐associated Weissellas (*W*. *confusa* and *W*. *cibaria*) (Lee et al., 2012; Muñoz‐Atienza et al., 2013; Patel et al., 2012). According to earlier research, the possibility of high natural resistance of *Weissella* species to quinolones (nalidixic acid), glycopeptides (vancomycin and teicoplanin), and aminoglycosides (except high‐level resistance encoded by aminoglycosides plasmidic modifying enzymes) can be found in different LAB species with a restricted horizontal transfer to other bacterial species (Ammor et al., 2007; Danielsen & Wind, 2003; Flórez et al., 2016; Imperial & Ibana, 2016; Jeong & Lee, 2015; Mathur & Singh, 2005; Toomey et al., 2010). Results of susceptibility and resistance for the tested antibiotics on the *Weissella* strains were consistent with previous studies (Akpınar Kankaya & Tuncer, 2020; Lee et al., 2012; Patel et al., 2012). The disk diffusion method can be applied for fast screening of strains, whereas the MIC assessment is the commonly accepted antibiotic resistance determination method for LAB.

Our results revealed that the incidences of chloramphenicol, tetracycline, and erythromycin resistance in the six strains were very low using the disc diffusion method. These findings were in line with studies declaring *Weissella* to be commonly susceptible to tetracycline, erythromycin, chloramphenicol, and ampicillin (Abriouel et al., 2015; Jeong & Lee, 2015). Remarkably, we noted very high MIC values of potential acquired resistance to erythromycin, tetracycline, and chloramphenicol, wherein we can find strains fully resistant to one or more clinically relevant antibiotics.

The antimicrobial‐resistant *Weissella* strains are belonging to *W*. *halotolerans*, *W*. *soli*, and *W*. *confusa*. In line with a recent study (Patrone et al., 2021), in *W*. *cibaria* strains we looked at, there was no indication of phenotypic antibiotic resistance. We noted that the EFSA Panel on Additives and Products or Substances used in Animal Feed (FEEDAP) et al. (2018) breakpoints for the genus *Leuconostoc* can be applied to the genus *Weissella* in the case of tetracycline.

The presence of antibiotic‐resistant LAB species is well documented for *Lactobacillus* and *Enterococcus* (Álvarez‐Cisneros & Ponce‐Alquicira, 2019; Hummel et al., 2007; Stefańska et al., 2021; Wang et al., 2019). Contrarily, there are limited data related to antimicrobial resistance for *Weissella* species and genetic determinants associated with antibiotic resistance (Muñoz‐Atienza et al., 2013; Basbülbül et al., 2015; Abriouel et al., 2015). In the present study, the susceptibility level of the strains to tetracycline is species and strain dependent. The most common tetracycline resistance mechanism is mediated by the *tet*(K) gene, encoding tetracycline efflux pump responsible for removing antibiotics to the outside of the cell, and followed by *tet*(S) and *tet*(M) genes, coding for ribosomal protection proteins, from tetracycline‐resistant *Weissella* strains. The Tet^r^ genes provided various levels of resistance to the *Weissella* strains. The *tet(O)* gene was detected only in a *W*. *confusa* V30 isolate, from plant, with MIC value of 32 mg/L. For the first time, our results revealed that the *tet*(K) gene for *W*. *soli* F96, as well as the *tet*(K) and *tet*(S) genes for *W*. *halotolerans* (FAS27), except for *tet*(M), were detected for tetracycline‐resistant *W*. *confusa* strains. A rare research revealed the *tet*(M) gene in *W*. *confusa* WCo‐1 (Abriouel et al., 2015). In most cases, enterococci carry frequently the *tet*(M) gene (Aarestrup et al., 2000). The tet(S) gene was initially discovered in *L. monocytogenes* strain BM4210 on a self‐transferable plasmid (Charpentier et al., 1993); however, it has been also reported on the chromosome of *L. mesenteroides* LbE16 strain (Flórez et al., 2016).

The *cat* and *ermB* genes were selected as they are the deeply studied and the most common spread resistance genes among LAB (Hummel et al., 2007; Thumu & Halami, 2012). The two *Weissella* strains that are resistant to chloramphenicol have high MICs (32 mg/L). There is a prospect that Weissella species have intrinsic resistance to chloramphenicol, which would limit the horizontal transfer of resistance to other bacteria. As regards the genetic basis of chloramphenicol (*cat*) and erythromycin (*ermB*) resistances could not be defined from the genomic DNA of the resistant strains, possibly due to the high variance of resistance genes. So, more investigations are requested to reveal the causal resistance mechanism. Muñoz‐Atienza et al. (2013) reported only Mef (A/E) drug efflux pump genes involved in the active efflux of macrolides in *W*. *cibaria* of aquatic origin. Nevertheless, *ermB* gene has been recently stated in *W*. *cibaria* DYE12 (Akpınar Kankaya & Tuncer, 2020). According to Abriouel et al. (2015), the lack of reports on the molecular identification of antibiotic resistance genes in Weissellas may be related to the significant variability in resistance genes.

The unsuccessful transfer of tetracycline resistance from *W*. *confusa* FS44 and FS63 to *E*. *faecalis* JH2‐2 as well as the vancomycin (data not shown) resistance might be attributed to a multitude of variables, including the use of inappropriate recipient strains and/or improper mating procedures. As a result, the tetracycline resistance of the two *W*. *confusa* was not transferred to the recipient strain in our investigation. Like *Leuconostoc*, no transconjugants were found when trying to transfer tetracycline resistance from *L. mesenteroides* to other bacteria (Toomey et al. (2010).

On the other side, the conjugation of *Bacillus thuringiensis* with the recipients *W*. *paramesenteroides* FS45, *W*. *confusa* (FS44 and FS63) strains, transconjugants was produced. With an average frequency of 3 × 10^−9^ transconjugants per receiver, transfer was low but detectable. This finding can be explained by the fact that pAW63 shares homology of Gram‐positive conjugation genes from *Enterococcus*, *Lactococcus*, *Listeria*, *Streptococcus,* and *Staphylococcus* species (Van der Auwera et al., 2005).

This is the first report to involve the antibiotic resistance determinants transfer of pAW63 (*erm*B) from *Bacillus thurigiensis,* which is ubiquitous in the environment, and closely associated with the food‐borne pathogen *Bacillus cereus,* potentially enterotoxigenic (Frederiksen et al., 2006).

These results led to suggest that *Weissella* is not a good vector to transfer antibiotic resistance genes, which can occur at a low frequency under laboratory conditions. It is a weak candidate to receive virulent determinants from closest gram‐positive pathogens. Consequently, further study incorporating mating settings is needed to assess the potential of *Weissella* spp. strains to spread antibiotic resistance.

As β‐hemolysis is linked to pathogenicity, in our investigation, tested *Weissella* strains did not exhibit hemolysis activity which is essential criteria for the selection of potential good strains.

Biogenic amines (BA) can be found in a variety of protein‐rich foods and fermented foods, and eating foods with excessive levels of these amines can generate toxicological consequences and health concerns (Durak‐Dados et al., 2020; Ruiz‐Capillas & Herrero, 2019; Santos, 1996). A variety of factors affect BA production, including the raw materials used, processing conditions, and microbes (Barbieri et al., 2019; Santos, 1996). The BA production in food by lactic acid bacteria has attracted a great interest and become the subject of considerable research because of their putative role in food poisoning (Barbieri et al., 2019; Ruiz‐Capillas & Herrero, 2019). Besides, the advantages of utilizing *Weissella* spp. as starter cultures and probiotics have recently received lots of interest (Gomathi et al., 2014; Kariyawasam et al., 2019). Interestingly, LAB belonging to *Leuconostoc* and *Weissella* genus are recognized as minor BA producers (Barbieri et al., 2019).

In this regard, phenotypic and molecular techniques should be used to investigate the occurrence of histidine, tyrosine, and ornithine decarboxylase activity in *Weissella* isolated from environment and animal sources. Strain‐level data concerning the ability for biogenic amine formation is requested to choose safe *Weissella* as starter for further food applications.

Up to now, there have only been a few reports on the role of some *Weissella* strains in the formation of biogenic amines. In this study, based on phenotypic analysis, 6 of 54 strains from environmental and animal origins produced one or more types of biogenic amines. Contrary to what Jeong and Lee (2015) reported, 44% of *Weissella* strains from Kimchi produced biogenic amines. For the first time, we reported tyramine production in two *W*. *halotolerans* (FAS29 and FAS30). *W*. *confusa* strains, from rhizosphere soil, have been found to generate putrescine, tyramine, and histamine. Regarding the production of biogenic amines in this species, these findings are consistent with earlier studies (Jeong & Lee, 2015; Takebe et al., 2016). Our study indicates that bacteria's main ability to decarboxylate amino acids is linked to their ecological niche from whence it originated as fermented foods, as well as strain specificity and amino acid decarboxylase gene diversity (Barbieri et al., 2019; Benkerroum, 2016; Jeong & Lee, 2015). Generally, these findings led us to suggest that the strains from environment and animal sources do not produce biogenic amines. In line with Garai et al. (2007) and Jeong and Lee (2015), we could suggest that this capability is strain dependent rather than species specific.

In this study, the level of inhibitory activity was greatly varied, depending on the tested *Weissella* strains. It was shown that the antagonistic activity against *E*. *faecalis* was found to be two times lower. This might be related to the fact that this species has a higher tolerance for pH and organic acids. The organic acids and other secondary metabolites of LAB were known to have inhibitory effect on growth. The neutralization of cell‐free supernatants and catalase treatment removed the antagonistic action against indicator bacteria. This demonstrates that a low pH environment and the presence of peroxide of hydrogen are the most important factors in preventing the growth of pathogenic bacteria.

The selected *W*. *confusa* (FS66, F77, and V28) and *W*. *halotolerans* (FAS17, FAS31, and V10) with good functional features and broad‐spectrum antibacterial activity throw up interesting perceptions as probiotic feed supplement in farm animals, notably in poultry, to prevent salmonellosis and colibacillosis. *W*. *confusa* (V5 and V31) and *W*. *halotolerans* V10, from desert plant, demonstrating the greatest anti‐*Staphylococcus* activity could be probiotic candidates on human and animal health.

Interestingly, *W*. *confusa* F77, from camel feces, was able to inhibit the growth of *Pa. larvae*, the causative agent of American Foulbrood of honeybees, a notifiable bacterial disease that destroys larvae of honeybees in many countries (Ebeling et al., 2016). Then, F77 showed suitable properties that make it good for its use as a probiotic in the honeybee diet. LAB has been shown to be important in controlling this disease by several studies (Daisley et al., 2020; Lamei et al., 2020; Mudroňová et al., 2011).

Therefore, the selection and availability of *Weissella* with good functional characteristics (such as antibacterial activity, lack of phenotypic and genetic virulence determinants, and no horizontal gene transfer) make them more attractive for potential applications as probiotics or technological candidates in food, feed complement, and agriculture. More research is needed to increase our understanding of enzymatic activities, metabolic systems in *Weissella* spp., suggesting the potential use of these strains as novel probiotics to reduce infection and limit antibiotic utilization, such as the prevention of intestinal infections in cattle production (Patrone et al., 2021). To our knowledge, this is the first large‐scale investigation detailing the antibacterial activity against numerous pathogens and the safety evaluation of *Weissella* spp. from diverse sources beyond the QPS procedure of LAB.

This is one of the few publications describing the characterization and probiotic potential of *Weissella* spp. from original sources. In this study, the in vitro assessment was performed to investigate the antibacterial activity against pathogens, the antibiotic susceptibility, the lack of transferable antibiotic resistance determinants, and the prevalence of virulence factors, which resulted in the selection of eight strains (five *W*. *confusa* and three *W*. *halotolerans*). This approach is a useful strategy for preliminary large‐scale selection of putatively safe *Weissella* strain for use as probiotics or supplements, as well as preventing the spread of bacterial cultures with harmful traits into the environment. Before the Weissellas can be considered recognizably safe probiotics, a full in vivo examination of their absence of cytotoxicity and undesirable effects must be carried out utilizing cell lines, raw food, and farm animals. Future investigations will be able to sustain the gained knowledge and assess the advantages.

## CONFLICTS OF INTEREST

The authors declare that there is no conflict of interests.

## ETHICAL APPROVAL

This study does not involve any human or animal testing.

## INFORMED CONSENT

Written informed consent was obtained from all study participants.
